# Chromite oxidation by manganese oxides in subseafloor basalts and the presence of putative fossilized microorganisms

**DOI:** 10.1186/1467-4866-12-5

**Published:** 2011-06-03

**Authors:** Magnus Ivarsson, Curt Broman, Nils G Holm

**Affiliations:** 1 Swedish Museum of Natural History, Department of Palaeozoology, Box 50007, SE-104 05 Stockholm, Sweden; 2 Stockholm University, Department of Geological Sciences, SE-106 91 Stockholm, Sweden

## Abstract

Chromite is a mineral with low solubility and is thus resistant to dissolution. The exception is when manganese oxides are available, since they are the only known naturally occurring oxidants for chromite. In the presence of Mn(IV) oxides, Cr(III) will oxidise to Cr(VI), which is more soluble than Cr(III), and thus easier to be removed. Here we report of chromite phenocrysts that are replaced by rhodochrosite (Mn(II) carbonate) in subseafloor basalts from the Koko Seamount, Pacific Ocean, that were drilled and collected during the Ocean Drilling Program (ODP) Leg 197. The mineral succession chromite-rhodochrosite-saponite in the phenocrysts is interpreted as the result of chromite oxidation by manganese oxides. Putative fossilized microorganisms are abundant in the rhodochrosite and we suggest that the oxidation of chromite has been mediated by microbial activity. It has previously been shown in soils and in laboratory experiments that chromium oxidation is indirectly mediated by microbial formation of manganese oxides. Here we suggest a similar process in subseafloor basalts.

## Background

Chromite, (Fe,Mg)(Cr,Al)_2_O_4_, is the primary geological source of chromium and is mostly concentrated in ultramafic rocks like peridotite and serpentinites. Chromite occurs sparsely in mafic rocks like basalts as an accessory mineral. Occasionally, chromite can be enriched due to early magmatic differentiation and occur as layers in mafic and ultramafic rocks as chromitite, a rock type that contains ~90% chromite. Chromite is a mineral with low solubility and is resistant to dissolution. Based on the thermodynamics, molecular oxygen, hydrogen peroxide and manganese (IV) oxides are capable of oxidizing Cr(III) to Cr(VI) at concentrations typically found in aquatic environments [1]. When considering the subsurface environment, direct Cr(III) oxidation by O_2 _is limited due to the slow kinetics [2]. Hydrogen peroxide production in the subsurface is limited and Cr(III) oxidation by this oxidant is probably insignificant. Mn(IV) oxides are, thus, the only known naturally occurring oxidants for Cr(III) [3-5]. It has been shown that the oxidation of Cr(III) is dependent and strongly accelerated by biogenic formation of Mn oxides [6-8]. Furthermore, chromium-bearing minerals like chromite have been shown to promote the abiotic formation of hydrocarbons in hydrothermal fluids [9,10].

It has recently been shown that fossilized microorganisms are present in subseafloor basalts. The most common morphological feature in deep drilled crust is granular and tubular ichno-fossils in volcanic glass [11,12], but body fossils have been recognised as well [13-18]. Both ichnofossils and body fossils are observed in or in association with veins and vesicles which the microorganisms use for migration through the rock. The veins and vesicles have at some stage been filled with secondary mineralizations like carbonates, clays or zeolites with the result of entrapment and entombment of the microorganisms.

**Figure 1:**
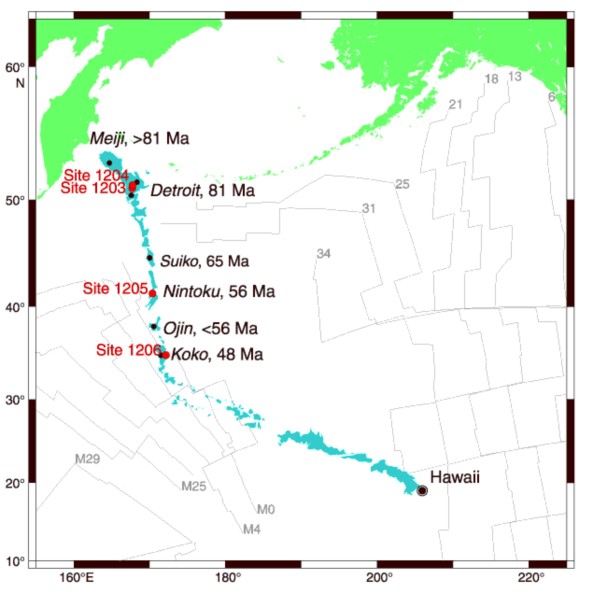
**Map showing the Emperor Seamounts and the sites drilled during ODP Leg 197**.

The Emperor Seamounts, which is a chain of submarine volcanic seamounts in the Pacific Ocean, were drilled during Ocean Drilling Program (ODP) Leg 197 at three different seamounts: Detroit, Nintoku and Koko seamounts, respectively (Figure 1). Ichnofossils and body fossils have been observed in drilled basalt samples from all three seamounts and the biogenicity of the fossilized microorganisms has been established by high amounts of carbon, hydrocarbons, phosphates, lipids and the presence of DNA [14-16]. In this paper we report observations made in one basalt sample: 197-1206A-37R-3, 72 cm, which was drilled and collected from Koko seamount (48 Ma) at a depth of 295 meters below seafloor (mbsf). In this sample we have a system of pentagonal and hexagonal chromite phenocrysts or pseudomorphs of the phenocrysts filled with Mn-carbonates, saponite and residues of the original chromite. These phenocrysts are all inter-connected by small carbonate or clay filled fractures, thus, at some point this local system of fractures and phenocrysts has been circulated by fluids. The carbonates are also rich in segmented filamentous structures similar to fossilized microorganisms previously observed in samples from this same site [14-16] as well as in a carbonate vein in this same sample [14].

Chromite oxidation by manganese oxides and microbial involvement have previously been studied in the context of Cr-contaminated soils and aquifers but neglected in subsurface or subseafloor settings with microbial connection. Here we will discuss the local micro-environment Mn oxidation of chromite gives rise to, and a possible connection with microbial activity.

### Geological setting

The submarine Emperor Seamount chain constitutes together with the Hawaiian Islands a continuous chain of volcanic seamounts and islands located at the center of the Pacific plate. The chain is considered to be the result of hotspot volcanism and extends over 5000 km [19]. The ages increase successively in a classical hotspot fashion to the northwest, away from the active hotspot. The youngest volcano that is related to Hawaiian volcanism today is the Loihi Seamount which is in the process of active formation. The ages of the Emperor Seamounts range from ~81 Ma to 43 Ma. From ~43 Ma to recent, the Hawaiian chain continues.

During Ocean Drilling Program (ODP) Leg 197, four Sites were drilled at three different seamounts; Detroit, Nintoku and Koko Seamounts. Sites 1203 and 1204 were drilled at the summit of Detroit Seamount and Site 1205 was drilled on Nintoku Seamount. Site 1206 was drilled on Koko Seamount at 1540 m of water depth with a final depth of 335.2 mbsf [19].

## Methods

In this study four doubly polished thin sections were produced from the same sample (197-1206A-37R-3, 72 cm) and used in the analyses. Efforts were made to avoid introduction of extraneous contamination. The thin sections were contained in aluminium foil, not touched with ungloved hands and only handled with stainless steel forceps. Mineral phases and filamentous structures were studied by a combination of microscopy, Environmental Scanning Electron Microscope (ESEM)/Energy Dispersive Spectrometry (EDS) and Raman spectroscopy.

The ESEM analyses were performed using a Philips XL 30 ESEM-FEG which is a field emission microscope. EDS analyses were performed using an Oxford x-act Energy Dispersive Spectrometer (EDS). The samples were subjected to a pressure of 0.5 torr and the accelerating voltage was 20 kV. The EDS analyses were performed by standardless quantification. A Back Scattered Electrons (BSE) detector was used and the penetration depth obtained was 0.5-3 μm.

The Raman spectroscopy analyses were performed using a Horiba Jobin Yvon LabRAM HR equipped with an Olympus BX41 optical microscope. We used a laser beam at 514.5 nm (green laser) with a precision of 1 μm. Detection was accumulated 10-20 times with measuring times ranging between 10-20 seconds. The obtained spectra were processed and identified by the software Labspec and RRUFF database.

## Results

### Mineralogy

**Figure 2:**
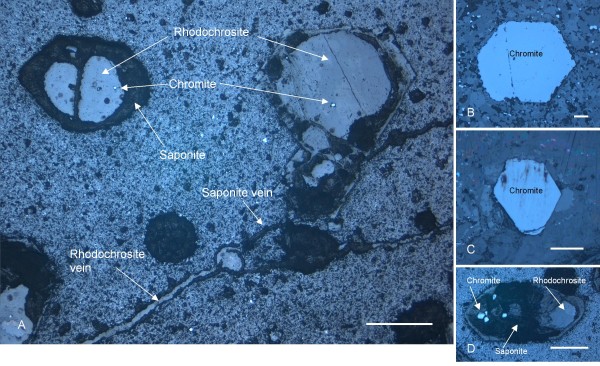
**Optical microphotograph obtained by reflective light**. (A) shows the phenocrysts and the mineral succession chromite-rhodochrosite-saponite therein. Veins of rhodochrosite and saponite connecting the phenocrysts are also visible. (B) and (C) show unaltered chromite phenocrysts and their hexagonal and pentagonal shape. (D) shows altered phenocryst with several chromite residues within. Scale bar: (A) 500 μm, (B) 100 μm, (C) 500 μm, and (D) 500 μm.

**Figure 3:**
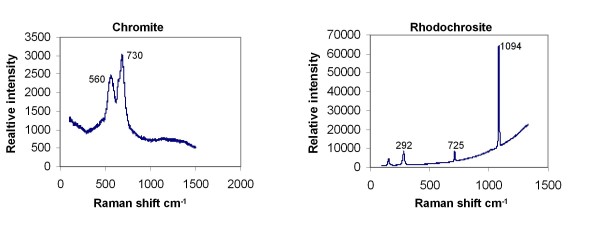
**Raman spectra of chromite and rhodochrosite**.

**Figure 4:**
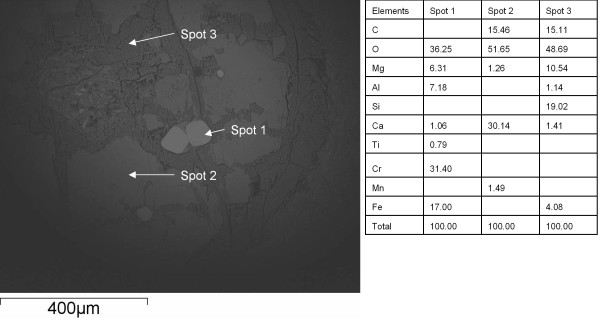
**ESEM microphotograph showing the mineral succession in the phenocrysts and EDS data for the minerals respectively**.

The pentagonal or hexagonal shape of the pseudomorphs (Figure 2) corresponds to the shape of unaltered chromite phenocrysts (Figure 2B,C). The pseudomorphs have been altered to various extent and consist of three phases based on Raman spectroscopy and EDS analyses (Figures 3 and 4): (1) Chromite, as the only, unaltered original phase or, most sparsely, as small grains which probably represent residues of the original mineral. (2) Rhodochrosite (MnCO_3_) as the dominating phase. Raman spectroscopy identified the carbonate phase as rhodochrosite but EDS analyses showed that the rhodochrosite is a Ca rich Mn carbonate. The Ca content can be as much as ~30 wt% and the Mn content is ~2-4 wt%. The rims of the rhodocrosite appear to have been weathered and replaced by (3) an unidentified clay phase. It is a heterogenous, slightly layered phase. EDS analyses showed that the phase contains Si, Al, Mg and Fe which corresponds to a smectite type clay, most likely saponite [20]. The pseudomorphs are all connected by minor veins which consist of saponite and rhodochrosite to some extent (Figure 2).

### Filamentous structures

**Figure 5:**
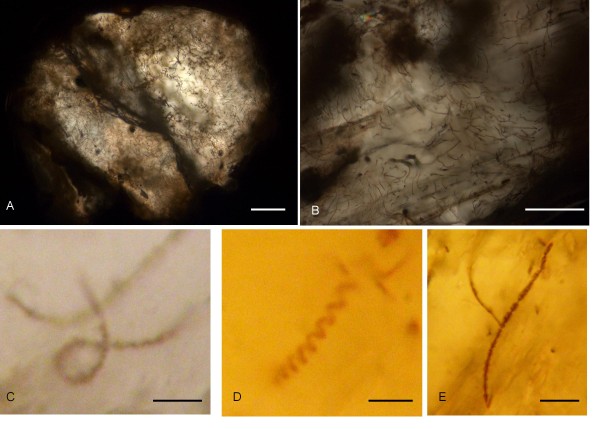
**Optical microphotographs showing the filamentous structures in the rhodochrosite**. (A) shows the abundance of filamentous structures in a rhodochrosite filled phenocryst. (B) Close up of filamentous structures showing their smooth, curvi-linear appearance. (C) microphotograph showing a filamentous structure coiled more than 360°. (D) microphotograph showing the twisted, spiral appearance. (E) microphotograph showing branching. Scale bar: (A) 100 μm, (B) 50 μm, (C) 5 μm, (D) 10 μm, (E) 10 μm.

**Figure 6:**
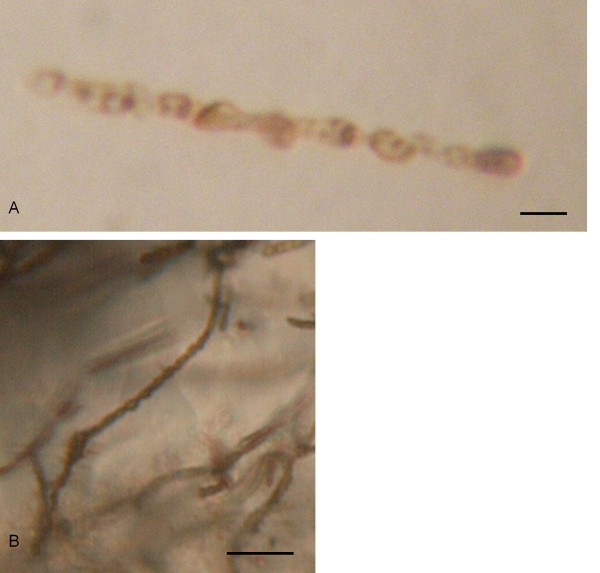
**Optical microphotographs showing the segmentation of the filamentous structures**. Scale bar: (A) 2 μm, (B) 10 μm.

Filamentous structures, 20-100 μm in length and ~1 μm in diameter, are found in the rhodochrosite-filled pseudomorphs of chromite (Figure 5). The appearance of these filamentous structures is uniform throughout all the rhodochrosite. They have a smooth, curvi-linear appearance that sometimes appears to be coiled. In a few cases the filamentous structures are coiled more than 360° (Figure 5C). A twisted, spiral appearance is also common and they branch frequently (Figures 5D,E). The filamentous structures have a distinct segmented appearance (Figure 6). Each segment is about 1-2 μm in diameter. They occur in large numbers and can more or less fill a whole rhodochrosite crystal (Figures 5A,B). Usually, the assemblages of filamentous structures are attached to the walls of the matrix but extend inwards into the vesicle in a complex network where the filamentous structures are intertwined in each other.

**Figure 7:**
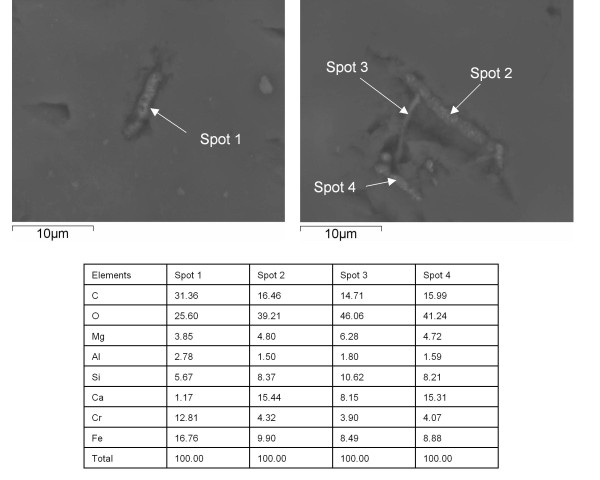
**ESEM microphotographs of filamentous structures in rhodochrosite and accompanied EDS data**.

EDS analysis showed that the filamentous structures consist of C, Fe, Si, Ca, Cr, Mg and Al (Figure 7). Due to the small size of the filamentous structures the EDS analyses might include some elements from the host mineral and the amounts of C and Ca can be influenced by the rhodochrosite. However, the presence of Si, Fe, Cr, Al and Mg indicate that the analyses are performed with a high degree of precision.

## Discussion

### Mineralogy

**Figure 8:**
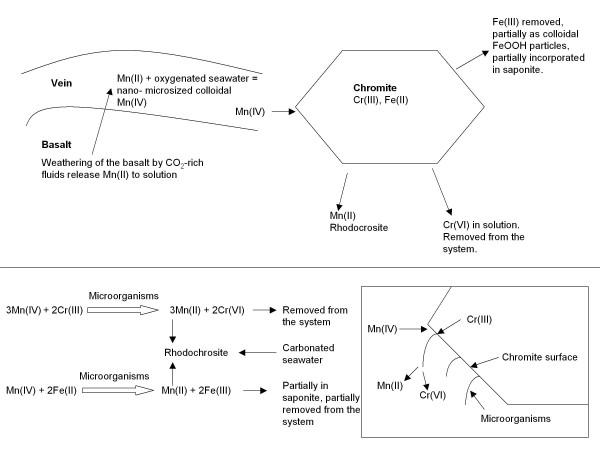
**Simplified sketch showing the main steps in the oxidation of chromites including weathering of the basalts, the influence of fluids, formation of carbonates, the possible involvement of microorganisms and above all the electron flow in the system, also illustrated by a redox equation**.

The paragenetic succession in the altered phenocrysts indicates that chromite is the primary mineral that has been replaced by rhodochrosite which in its turn has been replaced by a clay phase. Chromite is a common component in ultramafic rocks and occurs more sparsely in mafic rocks, but can be locally enriched due to mineral separation in the magma. Chromite is a mineral with low solubility and is thus resistant to dissolution. In fact, manganese oxides are the only known naturally occurring oxidants for chromite [3-8]. In the presence of Mn(IV) oxides, Cr(III) will oxidise to Cr(VI), which is more soluble than Cr(III), and thus easier to be removed by fluids (Figure 8).

The chromite in our samples was probably exposed to oxidised fluids containing micro- or nanosized colloidal Mn(IV)-oxides that was introduced into the system. The chromite was oxidized to Cr(VI) and subsequently removed from the system. The colloidal Mn(IV)-oxides were reduced to Mn(II), which is compatible with carbonates, and as fluids at carbonate saturation were introduced into the system the rhodochrosite was formed. Thus, the chromite was subsequently replaced by rhodochrosite and at a final step the rhodochrosite was weathered and partly replaced by a Fe-rich clay phase, interpreted as saponite. Chromite contains substantial amounts of iron which would have been oxidised as well during the oxidation of chromite. Oxidized iron is usually an element with limited mobility that tends to precipitate, however, iron oxides/oxyhydroxides are absent in the samples. One explanation could be that some of the iron was removed from the system as FeOOH particles. Another explanation could be that some iron may also have been incorporated in the saponite phase. The rate of carbonate formation exceeds the rate of Fe oxide formation thus it is also possible that Fe(III) was pushed out of the system.

In soils, oxidation of chromite by manganese is known to be influenced and controlled by pH, the amount and size of Mn oxides, and the presence of organic matter. Oze et al. [4] showed that Cr(VI) production increased with decreasing pH and that the production rate of Cr(VI) is limited in alkaline environments. The dissolution of Cr(III)- bearing silicates, on the other hand, is favored by alkaline conditions and the production rate of Cr(VI) increases with increasing pH. Organic material limits the production of Cr(VI) due to the reduction of Cr(VI) to Cr(III) in its presence [3]. Chung and Sa [3] further showed that a high pH and a low content of organic matter increases the chromium oxidation potential. Oxidation of chromite is also enhanced as the total surface area of Mn(IV) oxides is optimized. Nano- or microsized Mn oxide particles are likely to be more reactive than aged and well crystallized Mn oxides [6-8].

**Figure 9:**
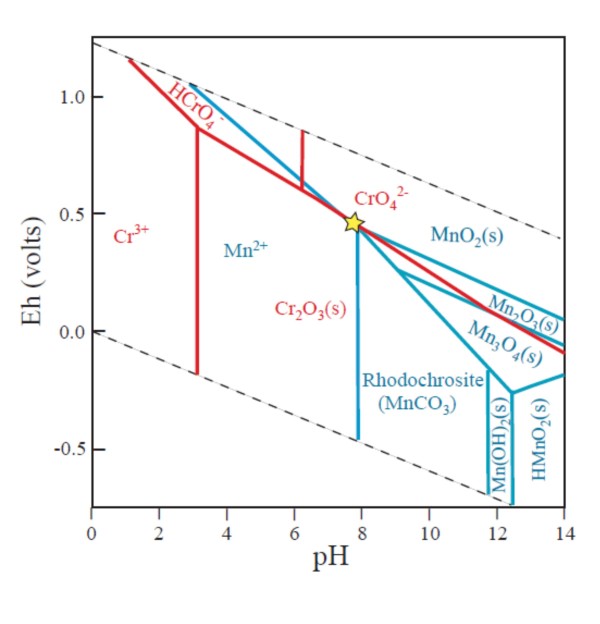
**Eh-pH diagram showing aquatic species and solid phases (s) of chromium and manganese at 25°C and 1 atm after data in **[37,38]. Red fields are for Cr and blue fields for Mn. Cr = 0.1 ppm, Mn = 0.1 ppm, HCO_3_^- ^= 100 ppm. The star indicates the system in the present study.

The pH of fluids in a basaltic rock is in general moderate, however, a system like the one in Sample 197-1206A-37R-3, 72 cm, that is characterized by hydrothermal activity and limited throughflow, is a dynamic system where localized fluctuations and steep gradients in the geochemical conditions will occur. Depending on mineral-fluid reactions, presence of organic matter or presence of microorganisms, pH can vary on a very local scale and it is thus difficult to discuss the conditions that prevailed while the chromite in our samples was oxidised and replaced. However, pH of seawater is neutral and the presence of carbonates suggests a pH around 7 or slightly higher. The Eh-pH diagram in figure 9 show that Cr(III) is oxidized to Cr(VI), and Mn(IV) is reduced to Mn(II) at pH 7. At pH 8 rhodochrosite is formed. This corresponds well to the suggested geochemical scenario of our samples.

### Putative fossilized microorganisms

The establishment of biogenicity of putative fossilized microorganisms is a complex task that commonly is subject to alternative interpretations. Several lists of criteria have been formulated to discriminate between abiotic and biotic structures [21-23]. Most of these criteria have been formulated from the study of microfossils in sedimentary rocks but Ivarsson [24] proposed a list of criteria adjusted to suit samples of crystalline rocks: (1) Is the geologic context compatible with life? (2) Is the putative microfossil indigenous with the rock (rather than being a modern contaminant)? Is the indigenous microfossil syngenetic with secondary minerals? (3) Does the sample contain evidence of microbiological morphology? (4) Does the fossil-like microstructure contain chemical remnants that are indicative of past life? Are any organic biomarkers present? (5) Is there evidence of structural remains of colonies or communities within the sample? (6) Is there any evidence of biominerals?

The following is an attempt to meet these criteria for the filamentous structures found in the rhodochrosite of Sample 197-1206A-37R-3, 72 cm. (1) Subseafloor settings have, during the last two decades, been shown to harbor a deep biosphere in both marine sediments [25] and basaltic basement [26-28]. Observations of microbial activity in the oceanic basement usually consist of granular or tubular ichnofossils in volcanic glass [11,12], but body fossils have also been reported [26,29,30]. Such microfossils have been associated with high amounts of carbon, phosphates, hydrocarbons as well as lipids and DNA [14-16]. Thus subseafloor basement is today recognized as a niche for microbial life and the geological context of our samples are compatible with life. (2) The rhodochrosite in the vesicles was formed from hydrothermal fluids circulating the basalt. As soon as the rhodochrosite formed, the putative microorganisms were trapped and preserved in the mineral. Thus, the putative microorganisms existed in the system contemporaneously with the hydrothermal activity, after the oxidation and weathering of the chromite but prior to the precipitation of rhodochrosite. (3) The morphology of the filamentous structures resembles known microbial morphology. The long, curvi-linear appearance with the segmentations is similar to known filamentous prokaryotes like, for instance, cyanobacteria [31]. The size of the single segments (~1-2 μm) also corresponds to known living prokaryotes. Branching is also common among microorganisms as is the coiled appearance. The twisted, spiral appearance that some of the filamentous structures display is common among certain microorganisms like Fe oxidising *Gallionella *sp [31]. Fossilized microorganisms from Detroit Seamount were shown to have similar spiral structure [15] and encrusted iron coated microorganisms collected from various hydrothermal vents display a twisted Gallionella-like morphology [32,33]. (4) We have not been able to detect any chemical biomarker that is indicative of life. The filamentous structures contain relatively high carbon values up to ~30 wt% in some samples. The C content in the filamentous structures is also higher or equal to the Ca, Fe and Mg content which indicates that the carbon is not bound in carbonates but originates from elsewhere. One possibility is that it is organic in origin, however, elevated carbon content is not an indication of past life nor can it be used as evidence for remnants of organic carbon. Other types of fossilized microorganisms, on the other hand, from the same core and the same sample have been interpreted as fossilized microorganisms and been shown to have high contents of organic remains as well as DNA [14,15]. (5) The filamentous structures occur without exception in abundant assemblages in the rhodochrosite. They occur in complex networks that resemble microbial mats [31], thus, we suggest that the filamentous structures represent remains of a microbial community. (6) The filamentous structures do not show evidence of encrustations on their cell surfaces or any type of associated biomineralisations which is typical among filamentous microorganisms and fossilized microorganisms from this type of environment [30,33]. However, the content of Fe and Cr could perhaps reflect a concentration of these elements in the cell walls which were preserved in the structures during the fossilization process. It could, however, also be the result of secondary incorporation of these elements from the surrounding environment during the fossilization process.

In our opinion, albeit possible to meet most criteria for biogenicity in a successful way except criterion no. 4. We lack sufficient chemical data to fulfil the criterion on organic remnants indicative of life. On the other hand, we believe that our results are in favor of a biogenic interpretation rather than an abiotic interpretation, thus we suggest that the filamentous structures should be regarded as putative fossilized microorganisms. Considering the amount of fossilized microorganisms that are present in the same samples [14,15] it is most likely that the structures in the rhodochrosite are fossilized microorganisms as well.

### Formation of Mn oxides, oxidation of chromite and the influence of microorganisms

In this paper we describe a local micro-environment clearly defined to merely a centimetre in the sample and dictated by specific geochemical conditions that happen to coincide (Figure 8). Mn(II) was first dissolved from the ocean floor basalts of the Emperor-Hawaiian hot spot during weathering by infiltrating seawater, followed by the formation of nano- or microsized (colloidal, <0.24 μm) Mn(IV) particles in contact with more oxygenated deep-sea water. Chromite exposed to fluids containing colloidal Mn(IV) particles was then available for autotrophic Cr-oxidising bacteria that used Mn(IV) as electron acceptor. The oxidized Cr(VI) was subsequently removed from the chromite mineral grain and replaced by rhodochrosite. This process results in a local chemistry of dissolved Cr(VI). Oxidized iron is usually an element with limited mobility that tends to precipitate. In these samples iron oxides are absent, but Fe occurs in the saponite phase, which is, therefore, probably syngenetic with the rhodochrosite. Very local chemical situations like this with steep redox gradients involve advantages for microorganisms. The putative fossilized microorganisms abundantly associated with the rhodochrosite contain substantial amounts of Cr and Fe. It is bold to discuss microbial metabolism based on element contents of fossilized microorganisms. The Cr and Fe content could be attributed to diagenetic effects of the fossilization processes or abiotic processes. However, it is worth noting that the only traces of Fe and Cr, except for the chromite residuals, are found in these filamentous structures. A possible scenario would be that a microbial community was nurtured by the dissolution of chromite. In this environment easy energy would be gained through oxidation of Cr(III) with Mn(IV) as electron acceptor, which would favor microbial activity.

Chromium oxidation in nature has been shown in several studies to be coupled with microbiological activity [6-8]. However, chromium oxidation is mostly indirectly mediated by microbial formation of manganese oxides. Wu et al. [6] showed in laboratory studies that Cr(III) oxidation was dependent upon the biogenic formation of Mn oxides and Murray and Tebo [7] showed in experiments that Cr(III) oxidation greatly accelerated in the presence of the Mn(II) oxidizing bacterium *Bacillus *sp. Strain SG-1. The Mn-oxides produced by the SG-1 were very reactive toward oxidizing Cr(III) and this was due to the small size and relative lack of order in the biooxides and the intermediates formed in the oxidation process. He *et al. *[8] compared in experiments the oxidation capacity of biogenic Mn oxide with three different Mn minerals (cryptomelane, todorokite, birnessite) with respect to Cr(III). They found that the oxidation capacity of biogenic Mn oxide was higher than all the mineral Mn oxides. Small particles or colloidal MnO_2 _have a high surface area and are less limited by diffusion, leading to increased probability of contact between Cr(III) and the oxidized Mn [34]. Nelson et al. [35] further showed that biogenic nanosized Mn oxides are likely to be more reactive in redox reactions than the well crystallized minerals. In soil samples Cr(III) oxidation is enhanced by "fresh and amorphous" Mn oxides. Aged and well crystallized Mn oxides, on the other hand, are weak and slow to oxidize Cr(III).

The putative microorganisms in our samples have not been Mn oxidizing microorganisms but more likely chromium oxidizing microorganisms using Mn(IV) as an electron acceptor. However, it is possible that Mn oxidising bacteria were involved in the conversion of leached Mn(II) to Mn(IV) during weathering of the basalts. Mn oxidizing microorganisms have been suggested to play an important role in the weathering and oxidation of subseafloor basalts and formation of ferromanganese crusts associated with hydrothermal vents. Thorseth et al. [25,29] have pointed out that that the frequent enrichment of Mn among encrusted microorganisms may indicate that Mn is an important energy source for subseafloor microorganisms at the Arctic Knipovich Ridge and the Australian Antarctic Discordance (AAD). Templeton et al. [36] identified 26 mesophilic, heterotrophic Mn-oxidizing isolates in weathered pillow basalts from Loihi Seamount. These bacteria were associated with Mn oxides at glassy margins of the pillow rims. The Mn oxides were also closely associated with and extensively intermixed with Fe oxides. Their interpretation was that Mn-oxidizing bacteria are directly dependent upon activity of chemolithoautotrophic Fe-oxidizing bacteria.

Whether the putative fossilized microorganisms in our samples have been directly involved in the oxidation of chromite or formation of the Mn carbonates or not, the environment with its redox potential is interesting in a microbial context. Chromite oxidation by manganese oxides has previously been discussed in the context of Cr-contaminated soils and aquifers but our results indicate that it can be an important process in subseafloor settings, not least in ultramafic hosted sites.

## Conclusions

In this paper we describe a system of interconnected pseudomorphs in a subseafloor basalt localised to Sample 197-1206A-37R-3, 72 cm drilled and collected during the ODP Leg 197 at the Koko Seamount. The mineral succession of the altered phenocrysts is chromite-rhodochrosite-saponite and we have interpreted it as the result of chromite oxidation by manganese oxides. The rhodochrosite contains abundant filamentous structures interpreted as putative fossilized microorganisms and we discuss the possibility of microbial involvement in the process of chromite oxidation and formation of rhodochrosite. The environment with its redox potential is interesting in a microbial context and could be of significance in mafic and ultramafic hosted subseafloor settings.

## Competing interests

The authors declare that they have no competing interests.

## Authors' contributions

MI carried out the analyses and drafted the manuscript. CB and NGH contributed to the interpretation of the results, the discussion and helped draft the manuscript. All authors read and approved the final manuscript.
